# Complete pathologic necrosis of a thyroid neoplasm following pressure-enabled thyroid artery embolization

**DOI:** 10.1210/jcemcr/luag246

**Published:** 2026-09-03

**Authors:** Sandra Gad, Nima Kokabi, Michael Mohnasky, Ralph P Tufano, Juan C Camacho

**Affiliations:** Department of Surgery, University of Miami, Miami, FL 33136, USA; Division of Vascular and Interventional Radiology, Department of Radiology, University of North Carolina at Chapel Hill, Chapel Hill, NC 27599, USA; School of Medicine, University of North Carolina at Chapel Hill, Chapel Hill, NC 27599, USA; First Physicians Group, Thyroid and Parathyroid Center, Division of Head and Neck Endocrine Surgery, Sarasota Memorial Health Care System, Sarasota, FL 34239, USA; Brian D. Jellison Cancer Institute, Sarasota Memorial Health Care System, Sarasota, FL 34239, USA; Department of Radiology, Radiology Associates of Florida, Sarasota, FL 34239, USA

**Keywords:** pressure-enabled drug delivery catheter, thyroid artery embolization, thyroid nodules, multinodular goiter (MNG), toxic goiter

## Abstract

Thyroid nodules are increasingly detected with advanced imaging. Although most are benign, accurate preoperative risk stratification remains essential. Fine-needle aspiration is central to evaluation, yet Bethesda III nodules remain challenging because of the variable malignancy risk. Thyroid carcinoma generally has a favorable prognosis, but certain molecular subtypes are associated with higher mortality. Therefore, surgical resection remains the standard treatment for indeterminate and malignant thyroid tumors. However, the thyroid's rich vascular supply, along with comorbidities and factors such as age and mediastinal extension, can increase operative risk. Preoperative transarterial embolization has emerged as an adjunct to reduce tumor vascularity and improve surgical outcomes. We report a case of a 72-year-old African American male with multiple comorbidities who presented with an indeterminate thyroid neoplasm harboring a *TERT* promoter pathogenic variant, indicating high malignancy risk. The patient underwent pressure-enabled thyroid artery embolization followed by thyroidectomy 72 hours postembolization. Lesion histopathology showed nuclear debris and ghost cells consistent with complete pathologic necrosis. This case supports the feasibility of embolization as an adjunct in the multidisciplinary management of high-risk patients and provides a foundational basis to further study embolotherapy in unresectable tumors or nonsurgical candidates.

## Introduction

With advances in imaging over recent decades, thyroid nodules are increasingly detected [1]. Although most nodules are benign, accurate preoperative risk stratification remains essential for management [1]. Fine-needle aspiration cytology, interpreted according to the Bethesda System for Reporting Thyroid Cytopathology, is central in evaluation and classification. However, Bethesda III nodules remain diagnostically challenging, as they are cytologically indeterminate and carry a variable, often underestimated malignancy risk of 23.5% to 40%, highlighting the limitations of cytology alone and the importance of molecular profiling to guide management. For example, telomerase reverse transcriptase (*TERT*) promoter pathogenic variants are associated with higher recurrence, local invasion, and mortality [2, 3]. When co-occurring with *BRAF* V600E pathogenic variants, *TERT* promoter pathogenic variants confer a worse prognosis due to synergistic activation of tumorigenic pathways [1, 3]. These molecular features have also been associated with resistance to radioactive iodine therapy, further complicating management [3].

Surgical resection is the standard of care for indeterminate and malignant thyroid tumors. However, due to the thyroid's rich vascular supply, surgery in large or invasive tumors carries a risk of intraoperative bleeding and operative morbidity [4]. In selected cases, preoperative transarterial embolization has emerged as an adjunctive strategy to reduce tumor vascularity, thereby lowering blood loss, facilitating safer resection, and allowing identification of critical structures [5-7]. The thyroid is supplied by the superior and inferior thyroid arteries (ITAs), which are interconnected via retrolobar anastomoses [8]. Traditionally, embolization requires selective catheterization of both arteries, which could be technically challenging and carries a risk of nontarget embolization, particularly during superior thyroid artery embolization due to possible reflux of particles into the internal carotid artery [9, 10]. Pressure-enabled thyroid artery embolization (PED-TAE) has recently been introduced as an alternative using ITA access, leveraging intrathyroidal collaterals to eliminate superior thyroid artery catheterization, potentially enhancing safety and reducing the risk of nontarget embolization.

Although embolization is well established in hypervascular malignancies, such as renal cell carcinoma and hepatocellular carcinoma (HCC), its application in thyroid cancer remains limited [11]. In HCC, bland arterial embolization is used as a bridging or downstaging therapy [12]. Notably, it has been associated with complete pathologic necrosis (CPN) rates as high as 60% [13]. However, its use outside the liver is uncommon.

We therefore present a case of a patient with a *TERT* promoter pathogenic variant indeterminate thyroid nodule who underwent pressure-enabled preoperative PED-TAE followed by thyroidectomy 72 hours postembolization to prevent blood loss and facilitate surgery [14]. Postoperative pathology demonstrated complete necrosis of the target lesion. This case supports the feasibility of embolization as an adjunctive preoperative strategy in selected patients with hypervascular thyroid malignancies and significant comorbidities. It also provides preliminary evidence to support further investigation of embolotherapy in primary thyroid cancer, particularly as a potential treatment option for patients who are not surgical candidates.

## Case presentation

A 72-year-old obese African American male with a history of hypertension and diabetes presented with a large multinodular goiter (MNG) and a 6-month history of progressive dysphagia and intermittent neck pain. On physical examination, there was marked anterior neck fullness with a diffusely enlarged thyroid gland. The mass was firm and immobile, without overlying skin changes or stridor at rest. The patient denied any family history of cancer or prior radiation exposure.

## Diagnostic assessment

At baseline, the patient was euthyroid (thyroid-stimulating hormone 0.495 mIU/L [SI: 0.495 mIU/L]; reference range, 0.550-5.00 μIU/mL [SI: 0.55-5.00 mIU/L]), free thyroxine (FT4) 0.89 ng/dL (SI: 11.45 pmol/L; reference range, 0.90-1.70 ng/dL [SI: 11.5-21.8 pmol/L]), free triiodothyronine 3.2 pg/mL (SI: 4.9152 pmol/L; reference range, 2.3-4.0 pg/mL [SI: 3.53-6.14 pmol/L]).

**Figure 1:**
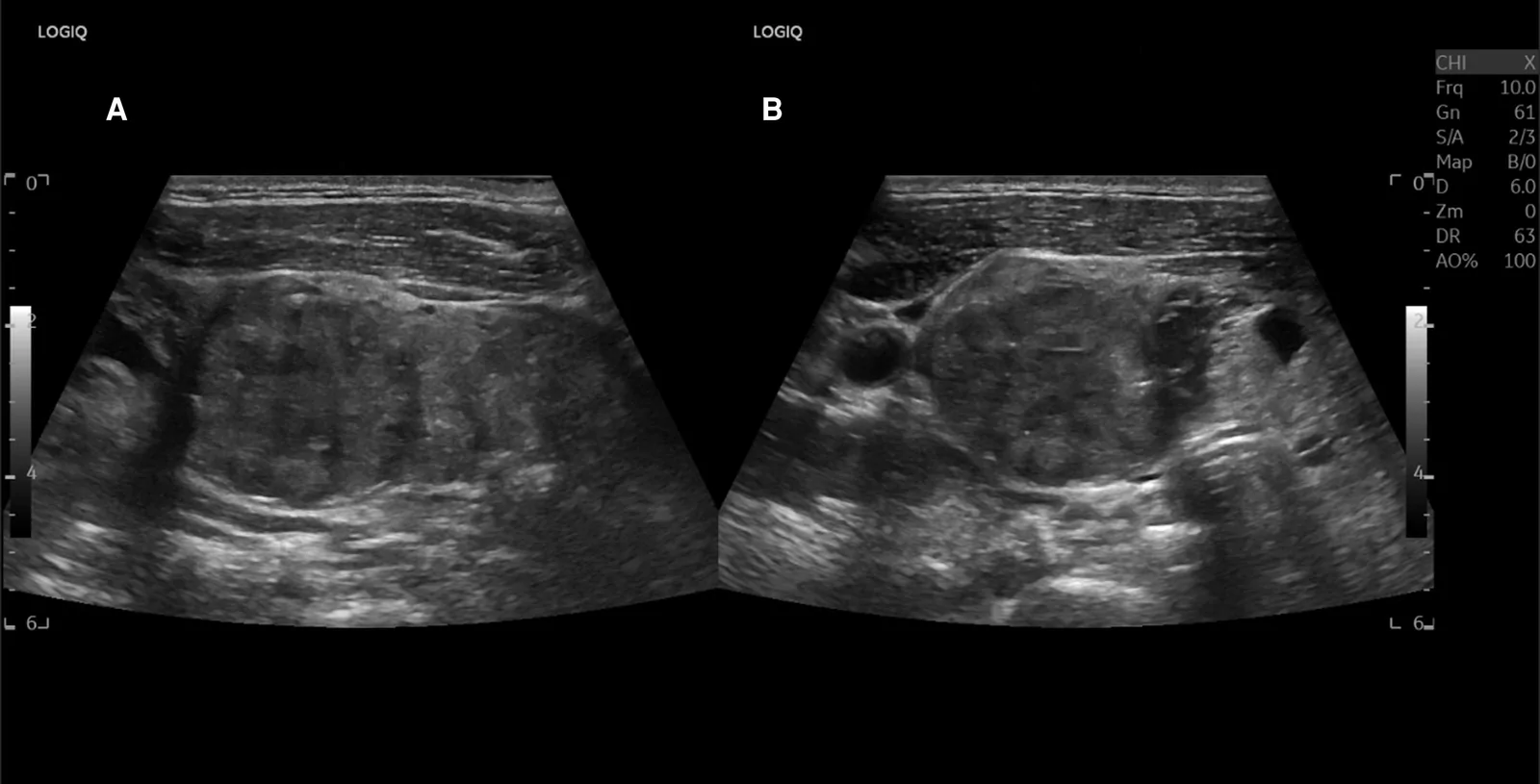
Ultrasound of the thyroid. (A) Sagittal view of the right lobe. (B) Sagittal view of the left lobe. Source: JC.

**Figure 2:**
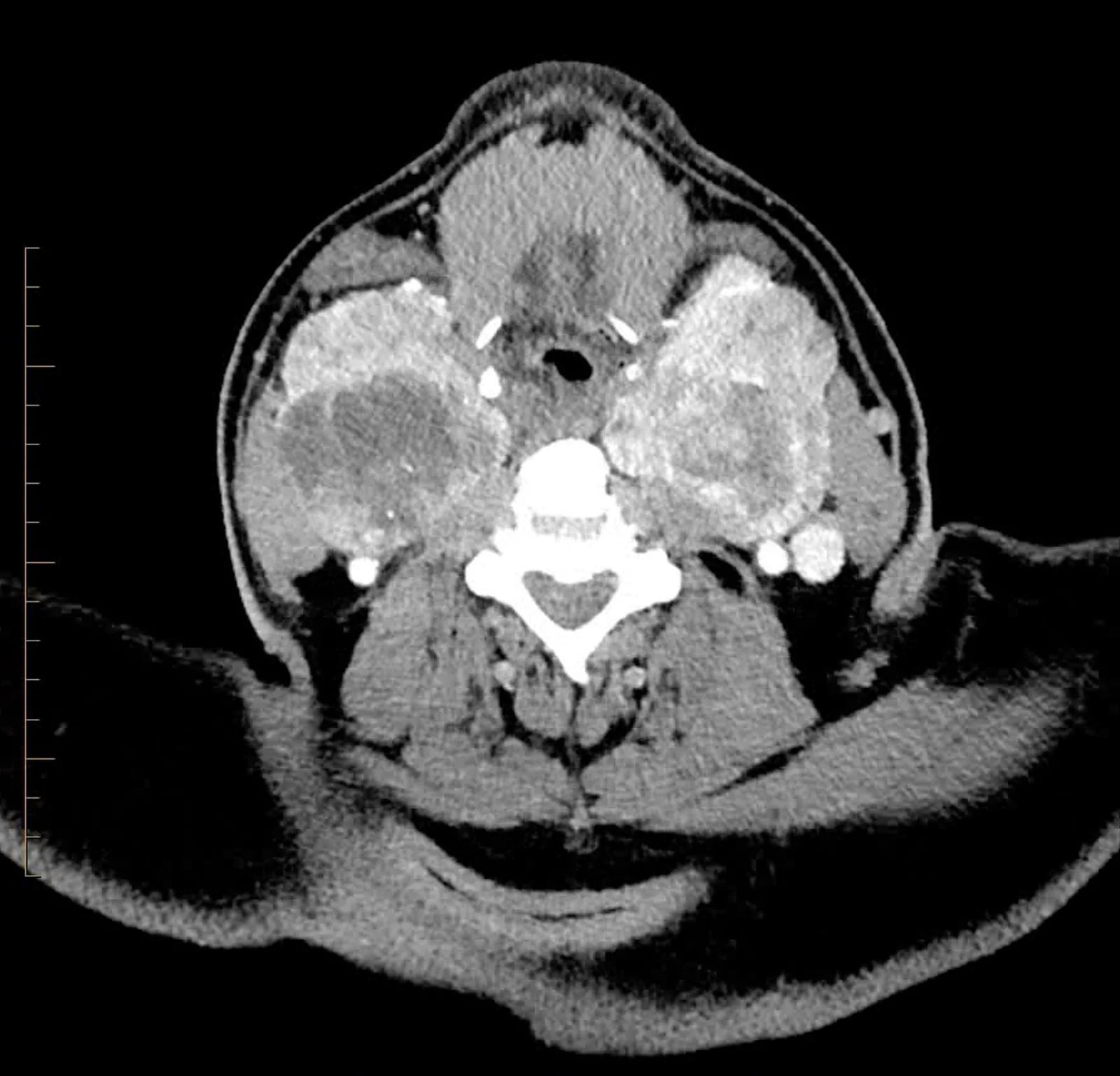
Computed tomography scan of the neck showed a diffusely enlarged thyroid gland, with the right lobe measuring 5.5 × 6.1 × 9.6 cm and the left lobe measuring 6.3 × 7.5 × 12.9 cm. Source: JC.

He underwent a thyroid ultrasound, which revealed a 4 cm round, encapsulated, hyperechoic, hypervascular solid lesion within the superior pole of the right thyroid lobe without definite microcalcifications, consistent with the American College of Radiology: Thyroid Imaging Reporting and Data System (ACR TI-RADS) 3 lesion, for which fine-needle aspiration is recommended (Fig. 1). Computed tomography (CT) scan of the neck showed a diffusely enlarged thyroid gland, with the right lobe measuring 5.5 × 6.1 × 9.6 cm and the left lobe measuring 6.3 × 7.5 × 12.9 cm, with a total gland volume of 465 mL. A barium swallow showed rightward deviation of the cervical esophagus from the mass effect of the thyroid, causing trace penetration without frank aspiration. Fine-needle aspiration of the suspicious right thyroid nodule, which showed atypical cells consistent with Bethesda III cytology. Molecular testing revealed a *TERT* promoter pathogenic variant with multiple chromosomal copy number alterations, conferring an estimated 80% risk of malignancy. The patient denied fever, chills, night sweats, and unintentional weight loss and had no additional pertinent medical history. Given the high malignancy risk associated with the *TERT* pathogenic variant, thyroidectomy was recommended (Fig. 2).

Due to the high anticipated risk of the surgical encounter and the potential for intraoperative bleeding, a multidisciplinary team, including surgery and interventional radiology, elected to proceed with preoperative PED-TAE. This decision was based on patient comorbidities and imaging findings, including a markedly enlarged (>200 mL), hypervascular thyroid gland with a large bilateral goiter and substernal extension [1, 2].

## Treatment

**Figure 3:**
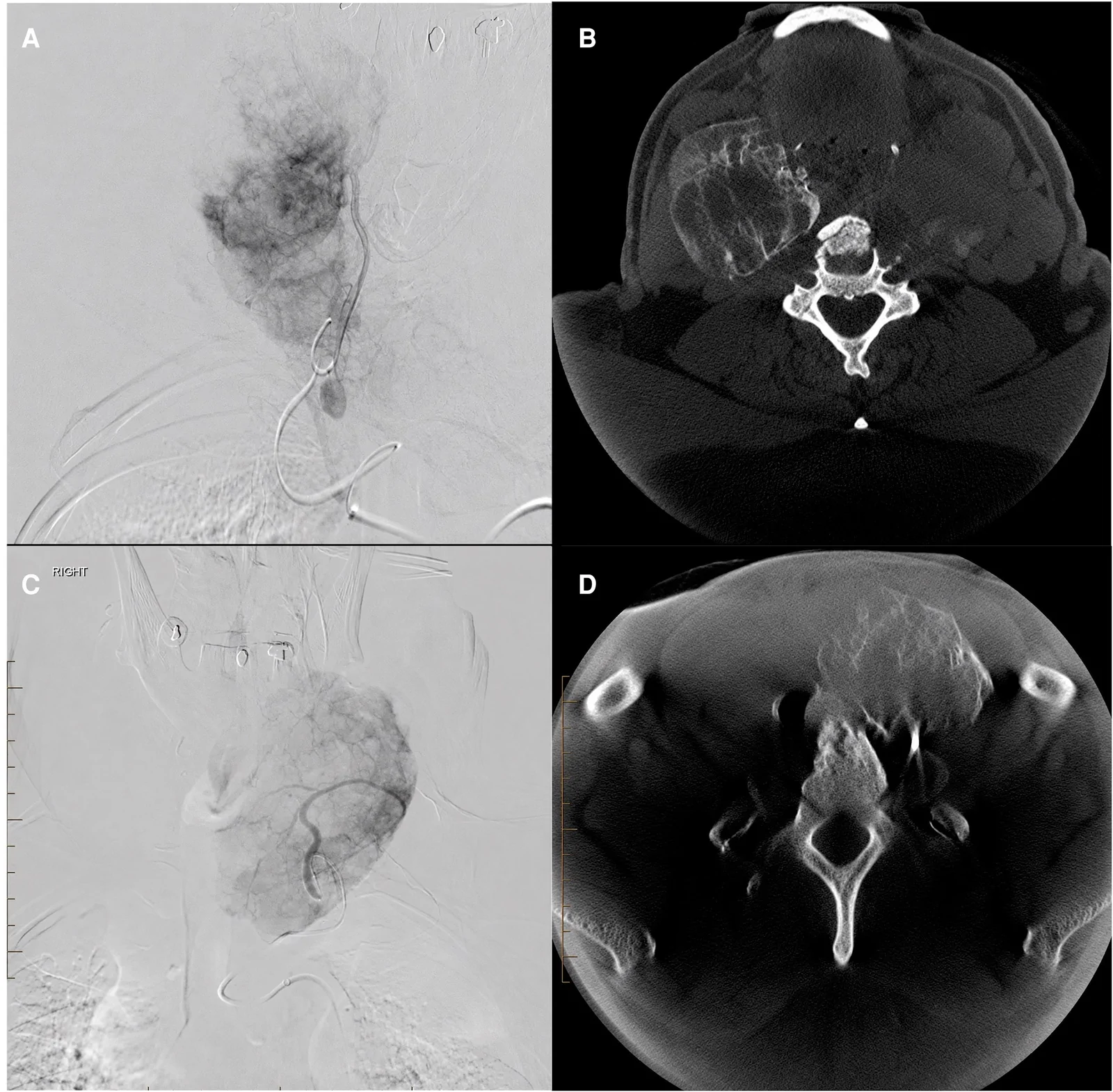
Inferior thyroid angiography with cone-beam CT correlation. (A) Angiography following catheterization of the left ITA with the pressure-enabled drug delivery device. Note the significant goiter hypervascularity without arterial reflux and opacification of the distal superior thyroid artery with flow reversal within the vessel. (B) Axial cone-beam CT on the angiography table demonstrating parenchymal enhancement from the catheter position for embolization without identification of vertebral or carotid branch collaterals, opacifying the whole left lobe from a single injection without catheterizing the superior thyroid artery. (C) Angiography of the right ITA with the pressure-enabled drug delivery device demonstrating significant goiter hypervascularity. (D) Axial cone-beam CT on the angiography table demonstrating parenchymal enhancement from the catheter position for embolization, opacifying the whole left lobe from a single injection without catheterizing the superior thyroid artery. Source: JC.

The patient underwent PED-TAE via right common femoral artery access under moderate conscious sedation. Diagnostic angiography demonstrated conventional thyroid arterial anatomy with prominent hypervascularity involving both thyroid lobes. No variant ITA or thyroid ima artery was identified. A pressure-enabled drug delivery system was then used for selective catheterization and embolic delivery. Embosphere microspheres (100-300 and 300-500 μm) were delivered through the left and right ITAs until near-stasis was achieved at each vessel. Postembolization angiography confirmed a significant reduction in thyroidal hypervascularity, with preservation of major cervical and vertebral artery branches. The procedure was technically successful and was completed without complications (Fig. 3).

## Outcome and follow-up

**Figure 4:**
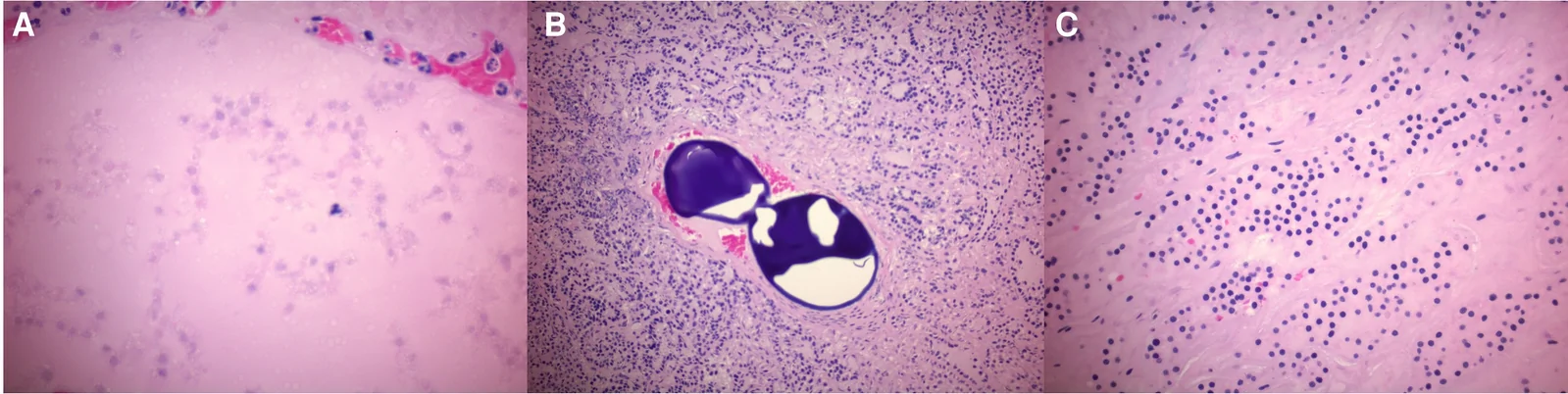
Microscopic findings of the resected thyroid mass showing complete necrosis (hematoxylin and eosin stain). (A) Frank necrosis (40×). (B) Intravascular bead within thyroid specimen (40×). (C) Ghost cells and inflammation of thyroid specimen (40×). Source: JC.

The patient subsequently underwent total thyroidectomy 72 hours postembolization with minimal intraoperative blood loss. The patient initially refused surgery due to severe anxiety on the same day of the embolization, hence the delay in surgery. Final pathology demonstrated CPN within the resected specimen confined to the neoplasm (Fig. 4). At follow-up, the patient remained clinically stable and euthyroid on replacement therapy, thyroid-stimulating hormone 0.05 mIU/L (SI: 0.05 mIU/L), FT4 1.6 ng/dL (SI: 20.6 pmol/L), and thyroglobulin (Tg) <0.2 ng/mL (SI: <0.2 μg/L; reference range, 2.8-40.9 ng/mL [SI: 2.8-40.9 µg/L]). FT4 is mildly elevated as expected due to levothyroxine therapy. Patient continues routine surveillance per American Thyroid Association recommendations. Surveillance includes measurement of a postoperative serum Tg level 6 to 12 weeks after total thyroidectomy and ultrasound every 1 to 3 years for 5 to 8 years, with discontinuation thereafter unless Tg rises or Tg antibodies become newly detectable.

## Discussion

With advances in technology and imaging modalities, the detection of thyroid cancer has increased over recent decades, with Bethesda III nodules accounting for up to 25% of thyroid fine-needle aspiration biopsies in the United States [15]. A *TERT* promoter pathogenic variant in a Bethesda III nodule confers a high-risk of malignancy (∼65-93%) and is considered a high-risk molecular feature that typically warrants surgical intervention, as these tumors exhibit more aggressive behavior and are associated with poorer clinical outcomes [3, 4]. Standard surgical treatment can be challenging at times, as the thyroid gland's hypervascularity, combined with patient comorbidities and modifiable conditions such as obesity, can increase surgical complexity and the risk of postoperative morbidity, including wound complications, venous thromboembolism, acute kidney injury, shock, and cardiac events [4, 16]. Postoperative mortality has also been reported, particularly in cases complicated by bleeding requiring reintervention, postoperative hematoma, and/or incomplete resection [4, 16]. Notably, the presence of 3 or more comorbidities has been associated with mortality rates as high as 12.5% [4, 16].

Transarterial embolization may be beneficial in such high-risk cases prior to resection and is well established in other hypervascular tumors, such as HCC and renal cell carcinoma; however, data on its role in thyroid cancer remain limited [6-8]. The conceptual rationale is similar, with the goal of achieving devascularization of the tumor preoperatively, thereby reducing intraoperative hemorrhage and improving visualization of tissue planes [11, 17, 18]. In addition, the benefits of thyroid embolization observed in patients with MNG may extend to nonsurgical or high-risk surgical candidates, as TAE has demonstrated greater efficacy than ablation in reducing thyroid volume and relieving compressive symptoms [6, 7, 14, 19]. Therefore, in the thyroid malignancy context, potential candidates for preoperative TAE may include select patients with large, hypervascular thyroid tumors, substantial compressive symptoms, or anticipated operative challenges due to bleeding risk; however, formal indications remain to be defined. Currently, preoperative TAE should be considered within a multidisciplinary framework, and TAE in thyroid malignancy was first described in a case report roughly 40 years ago [20]. Nearly 2 decades later, it was reported as a treatment option for locally advanced thyroid cancer [21]. Since then, several studies and case reports have documented its use in thyroid malignancies [9, 21-24]. The largest study to date included 13 patients who underwent preoperative TAE, aiming to reduce tumor size either for resection or as palliative treatment [9]. Minor complications, such as postembolization syndrome and hematoma, were observed. However, no major complications were observed, and patients experienced significant symptom relief [9].

All prior studies used a traditional approach requiring catheterization of both the inferior and superior thyroid arteries, which could increase nontarget embolization related complication. In contrast, the present case utilized a pressure-enabled delivery device via ITA access, leveraging intrathyroidal collaterals to treat the superior thyroid territory [8]. This single-access approach may reduce the risk of nontarget embolization and cerebrovascular complications [25]. A recent retrospective study supports its safety and feasibility in patients with large MNGs, demonstrating significant volume reduction [14]. Pressure-enabled delivery may allow more uniform and distal embolic penetration, enhancing devascularization and potentially contributing to the CPN observed in this case, which we also believe was influenced by the delay in surgery [14].

It is very important to mention that formal indications and patient selection criteria for TAE in thyroid malignancy remain undefined, and its use should be considered within a multidisciplinary framework. Currently, embolization is primarily used for palliation, with no established role as a definitive therapy. General contraindications include renal insufficiency, contrast allergy not amenable to premedication, and unfavorable vascular anatomy, none of which were present in this patient. In this case, embolization was followed by thyroidectomy within 72 hours with minimal blood loss. Although surgery is ideally performed within 24 hours to limit potential inflammatory and adhesive changes, the impact of delayed resection remains uncertain and warrants further study [21].

An additional point that warrants discussion is the histology of the surgical specimen, which demonstrated CPN (Fig. 4). Complete pathologic necrosis indicates that no viable tumor cells remain in the examined specimen, signifying a maximal response to treatment [26]. Treatment-induced CPN is considered an independent positive prognostic factor in patients with a number of malignancies, such as bone sarcomas and HCC [27, 28]. This case demonstrates CPN following embolotherapy as an adjunct to surgery in thyroid malignancy. While its prognostic significance in thyroid malignancy remains undefined, this raises the potential for long-term benefit and the hypothesis that embolization should be explored as an alternative in patients unable to undergo standard of care therapy. The successful outcome in this case highlights the value of a multidisciplinary approach. Close collaboration among interventional radiology, endocrine surgery, and pathology enabled preoperative planning and effective surgical resection. This model mirrors the approach used in hepatobiliary oncology, where multidisciplinary tumor boards routinely integrate embolization into treatment strategies for complex tumors. Given similar vascular anatomy and surgical considerations, a comparable approach may benefit thyroid oncology.

Despite the encouraging findings, several limitations exist, including the single-case design, lack of long-term oncologic outcomes, lack of definitive pathological analysis of the nodule, absence of follow-up imaging, and potential risks associated with TAE. Cost-effectiveness and availability were also not assessed. Prospective multicenter studies are needed to better define the role of embolization in thyroid cancer. Nonetheless, this case suggests that embolization may have a role within a broader multidisciplinary strategy for selected high-risk patients, as well as providing foundational knowledge to study embolotherapy for unresectable thyroid malignancies.

## Learning points

- Integration of embolization as part of a multidisciplinary approach in the management of thyroid tumors may improve patient outcomes.
- Preoperative PED-TAE may reduce vascularity and facilitate resection in select patients with large, hypervascular thyroid tumors.
- Histopathology of thyroid neoplasm specimen displayed CPN, a finding associated with improved outcomes after neoadjuvant therapy in multiple cancers.
- Complete pathologic necrosis observation supports further investigation of PED-TAE as an alternative for patients without surgical options, with a focus on the reproducibility of necrosis and its correlation with oncologic outcomes.

## Contributors

All authors made individual contributions to authorship. J.C.C. and R.P.T. were involved in the diagnosis and management of the patient. S.G., N.K., and M.M. were involved in manuscript preparation and data collection. All authors reviewed and approved the final draft.

## Data Availability

Original data generated and analyzed during this study are included in this published article.
